# Fibrinogen was associated with subgingival microbiome in periodontal diseases: a pilot study

**DOI:** 10.1080/20002297.2026.2681264

**Published:** 2026-06-02

**Authors:** Aopeng Zhang, Ruolan Du, Lei Lei, Jianan Liu, Gao Sun, Rui Wang, Tao Hu, Ran Cheng

**Affiliations:** a State Key Laboratory of Oral Diseases & National Center for Stomatology & National Clinical Research Center for Oral Diseases & Frontier Innovation Center for Dental Medicine Plus & West China Hospital of Stomatology, Sichuan University, Chengdu, Sichuan, People's Republic of China; b West China Hospital of Sichuan University-Ziyang Hospital & Ziyang Central Hospital, Ziyang, Sichuan, China; c GeneScience Pharmaceuticals Co., Ltd., Changchun, People's Republic of China; d Hospital of Stomatology, Jilin University, Changchun, People's Republic of China

**Keywords:** Gingivitis, periodontitis, gingival crevicular fluid, fibrinogen, oral microbiome, 16S rRNA sequencing

## Abstract

**Background:**

Periodontal diseases are associated with complex interactions among inflammatory responses, microbial dysbiosis, and clinical periodontal parameters. However, the relationships among inflammatory biomarkers, microbial biomarkers, and clinical parameters in gingival crevicular fluid (GCF) remain to be further clarified.

**Objective:**

To explore the interactions among inflammatory biomarkers, microbial biomarkers, and clinical parameters in gingival crevicular fluid in periodontal diseases.

**Design:**

In this cross-sectional study, GCF and subgingival plaque were collected from healthy, gingivitis, and periodontitis participants. Levels of fibrinogen, fibrin (measured as fibrin degradation products (FDP)), Interleukin (IL)-1β, IL-17, Matrix metallopeptidase (MMP)8, and MMP9 were measured by enzyme-linked immunosorbent assay. The subgingival microbiome was analyzed using 16S rRNA gene sequencing.

**Results:**

Among all biomarkers, fibrinogen was the most sensitive biomarker detected in GCF. Levels of fibrinogen were higher in the gingivitis (*p *= 0.024) and periodontitis (*p *= 0.002) groups than in the healthy group. Positive correlations were found between fibrinogen and numerous subgingival microorganisms, such as *Tannerella forsythia*, *Treponema denticola*, *Porphyromonas gingivalis*, and *Filifactor alocis*. Fibrinogen was the only GCF marker that could differentiate between healthy and gingivitis individuals. Fibrinogen and its combination with specific subgingival microorganisms may be potential markers indicating gingivitis and periodontitis.

**Conclusions:**

Compared with IL-1β, IL-17, MMP8 and MMP9, fibrinogen in GCF demonstrated distinct associations with clinical parameters and subgingival microbiome in periodontal diseases.

## Key messages

- Fibrinogen is correlated with periodontal parameters and subgingival microbiome.
- Fibrinogen can distinguish periodontal health from gingivitis and periodontitis.
- Subgingival microorganisms combined with fibrinogen improves diagnostic accuracy.

## Introduction

Periodontal diseases are inflammatory diseases influenced by a variety of factors. Gingivitis and periodontitis are two common types of periodontal diseases, of which gingivitis is reversible, but tooth loss and alveolar bone resorption due to periodontitis are irreversible [1]. With an estimated 796 million people worldwide affected by severe periodontitis, periodontal diseases remain a significant global disease burden and population health challenge [2]. Dysbiosis, an imbalance in the oral microbiome, is considered to be the main aetiologic factor of periodontal diseases [3]. The colonisation and composition of oral microbial communities are affected by inflammatory responses, diet, and oral hygiene habits [1]. In microbial dysbiosis, multiple microorganisms interact to increase colonisation and produce a sustained inflammatory response in periodontal tissues [4]. Inflammation can affect host defence and alter the local environment [5], promoting the growth of inflammophilic pathobionts [6]. A mutually beneficial causal association seems to exist between inflammation and the microbiome [7,8]. However, the relationship between the two requires further investigation.

The ‘red complex’, proposed in the microbial complex framework by Socransky et al., refers to a group of periodontitis-associated taxa, classically including *Porphyromonas gingivalis* (*P. gingivalis*), Tannerella forsythia (*T. forsythia*), and Treponema denticola (*T. denticola*) [9]. Some new species, such as *Filifactor alocis* (*F. alocis*), were also found in periodontitis [10][11]. Significant differences have been found in the subgingival microbiome in health or disease conditions [12]. The microbial and inflammatory profiles have been detected in periodontally healthy individuals and different stages of periodontitis. The red complex species, orange complex species and higher IL-1β/IL-10 ratio were detected in aggressive periodontitis than in healthy individuals [13] . The microbiota composition and cytokine profile changes in patients with stage III/IV periodontitis (Stage III involves 50% bone loss, probing depths 5 mm, and potential furcation involvement, whereas Stage IV represents advanced disease with severe destruction, tooth mobility) were also demonstrated, with more *Synergistetes*, *TM7*, *SR1*, *Spirochaetes*, *Bacteroidetes*, *Fusobacteria*, IL-1β, IL-4, IL-10, and IL-17A detected in the diseased sites [14]. However, the interaction between microbiota composition and cytokine profile would be potentially meaningful, which requires further investigation.

Diagnosis of periodontal diseases requires the detection of clinical parameters and radiographic examinations. Nevertheless, the clinical diagnosis only reveals the presence of disease and has limited prognostic capabilities. Biochemical biomarkers of periodontal diseases may have the potential for screening and disease discrimination [15]. Various proteins, including IL-1β and MMP8, present in saliva or GCF, are currently recognised as potential biomarkers for the initiation and progression of periodontal disease [16–20]. In our previous research, multiple microorganisms, including *F. alocis*, *Campylobacter gracilis* (*C. gracilis*), , and *P. gingivalis*, demonstrated positive correlations with clinical parameters, suggesting the potential of microorganisms as risk indicators for monitoring periodontal diseases [21]. Single biomarkers and a combination of multiple biomarkers have been a long‐term approach in assessing periodontal diseases [22]. The combined assessment of related inflammatory and microbial biomarkers may show promising diagnostic or prognostic abilities.

In this study, we aimed to explore the interaction among inflammatory biomarkers, microbial biomarkers, and clinical parameters in healthy, gingivitis, and periodontitis individuals. The discriminating efficacy of single and combined inflammatory and microbial biomarkers was assessed accordingly.

## Materials and methods

### Study design and participants

This cross-sectional study was conducted in accordance with the Declaration of Helsinki and Chinese clinical trial standards. All relevant information was reported according to the Strengthening the Reporting of Observational Studies in Epidemiology (STROBE) and the Standards for Reporting of Diagnostic Accuracy Studies (STRAD) guidelines. The study was approved by the Ethics Committee of West China Hospital of Stomatology, Sichuan University (WCHSIRB-D-2022-271) and registered in the Chinese Clinical Trial Registry (ChiCTR2200059339). Participants were recruited at the Department of Preventive Dentistry, West China Hospital of Stomatology, Sichuan University from July 2022 to November 2022. Sixty consecutive subjects were recruited from a convenience sample seeking dental care at the hospital. All subjects between the ages of 18 and 65 were invited to participate and were informed of the associated risks and benefits before providing informed consent.

G*Power (v3.1.9.7, Heinrich Heine University, Düsseldorf) was used to calculate the sample size. To detect an effect size of 0.45 with 85% power at *α* = 0.05, each group needed at least 20 participants [21].

Periodontal health and disease diagnosis was performed according to the 2017 World Workshop on the Classification of Periodontal and Peri-Implant Diseases and Conditions [23–25]. Participants with bleeding on probing (BOP) <10% and with probing depth (PD) ≤3 mm were categorised into the healthy group. The gingivitis group consisted of individuals with BOP ≥10% and probing pocket depths (without pseudo pockets) ≤3mm. Patients with interdental clinical attachment loss (CAL, detectable at ≥2 non-adjacent teeth), or buccal/oral CAL ≥3mm with pocketing >3mm (detectable at ≥2 teeth) were assigned to the periodontitis group. Patients with periodontitis were staged according to the degree of CAL and tooth loss [25]. Exclusion criteria were as follows: pregnancy or lactation; the presence of systemic diseases such as diabetes; smoking; having received professional periodontal treatments within the previous 3 months; having taken antibiotics or any other medication within 3 months; xerostomia or oral tumours; use of orthodontic appliances or removable dentures.

### Clinical examinations

A full mouth periodontal examination was performed by a single examiner and was used as the standard for periodontal health, gingivitis, and periodontitis diagnosis. The examiner was calibrated by an experienced dentist before patient examination. All participants underwent a 4-site periodontal examination of each tooth (mesio-labial/buccal, mid-labial/buccal, disto-labial/buccal, and lingual/palatal) to record the plaque index (PI) and modified gingival index (MGI). A computerised periodontal probe (Florida Probe^®^ - Florida Probe Corp., FL, USA) was used to examine PD, CAL, and BOP at six sites per tooth (mesio-buccal, buccal, disto-buccal, mesio-lingual, lingual and disto-lingual). MGI, PI, BOP, PD, and CAL values were evaluated in all the teeth of the participants.

### Samples collection

One week after clinical examination, the GCF and subgingival plaque samples were collected between 8 and 10 a.m. Participants were required to suspend all oral hygiene measures for 12 hours and prohibited from drinking or eating for at least 2 hours before giving their sample. The samples were collected from the mesio-buccal, buccal, and disto-buccal of single-rooted teeth (the incisors, canines, and premolars) in each individual. Following the isolation of the selected sites, the tooth surface was air-dried and the supragingival plaque was removed. The samples were collected by filter paper strips. The strip was gently inserted into the gingival crevice or periodontal pocket and held there for 30 seconds. Samples with visible blood were discarded. Subgingival plaque samples were stored at –80 °C until further analysis. For GCF samples, the strips were placed into a storage tube containing 500 μL phosphate-buffered saline (PBS) and centrifuged at 1000 × g for 10 min at 4 °C. The supernatant was collected and transferred to a –80 °C refrigerator for storage.

### Characterisation of inflammatory biomarkers and subgingival plaque

The levels of IL-1β (pg/mL), IL-17 (pg/mL), MMP8 (ng/mL), MMP9 (ng/mL, all MultiSciences, Hangzhou, China), fibrin (measured as fibrin degradation products (FDP), μg/mL, MyBioSource, Cat. NO. MBS2022486, San Diego, CA, USA), and fibrinogen (ng/mL, Abcam, Cambridge, UK) in the GCF were measured using commercially available enzyme-linked immunosorbent assay (ELISA). Subgingival microbial DNA was extracted using ZymoBIOMICS DNA Microprep Kits (Zymo Research, Irvine, CA, USA) following the manufacturer’s instructions. Amplified the full-length fragment of the microbial 16S rDNA gene using specific primers 8F (5'-AGAGTTTGATCATGGCTCAG-3') and 1492R (5'-CGGTTACCTTGTTACGACTT-3'). Analysed the amplified products by agarose gel electrophoresis, and recovered and purified the samples that pass the detection. Constructed a gene library and performed high-throughput sequencing, then used NanoFilt for data quality control. Completed species annotation using Kraken2 and constructed an operational taxonomic units (OTU) table for downstream analysis.

### Statistical analysis

The data were analysed using SPSS statistical software. Basic demographic information was assessed using descriptive statistics, with categorical variables expressed as frequencies and percentages, and continuous variables expressed as mean and standard deviations, or median and quartiles. Kruskal-Wallis test was used to compare the differences between groups, and multivariate logistic regression analysis was performed to explore the effects of inflammatory biomarkers on the occurrence of periodontal diseases. For multiple comparisons of the intergroup differences, *p*-values were considered significant after the Bonferroni correction (*p* < 0.05) to control the family-wise error rate. To explore the associations between subgingival microbiome and clinical/inflammatory factors, Spearman rank correlation analyses were restricted to the top 50 most abundant subgingival species due to the high dimensionality of microbiome data. For these correlation analyses, *p*-values were adjusted using the Benjamini–Hochberg false discovery rate (FDR) procedure to control the false discovery rate, with statistical significance defined as an FDR-adjusted *p*-value < 0.05.

Determined the receiver operating characteristic (ROC) curves for the markers and calculated the area under the curve (AUC). Significant inflammatory factors and subgingival microorganisms were selected based on the results of nonparametric tests. Composite ROC curves were generated by combining the inflammatory biomarkers and subgingival microorganisms in a logistic regression model. AUC values were interpreted as low diagnostic accuracy (0.50–0.70), moderate diagnostic accuracy (0.71–0.90), and high diagnostic accuracy (>0.90) [18]. Diagnostic measures also include sensitivity, specificity, positive predictive value (PPV), and negative predictive value (NPV). The optimal diagnostic threshold was determined by calculating the Youden Index (YI).

## Results

### Characteristics of the participants

A total of 60 participants were included in this study, including 20 individuals in each of the healthy, gingivitis (10% ≤ BOP% < 30%: *n* = 18; BOP% ≥ 30%: *n* = 2), and periodontitis (localised stage Ⅰ: *n* = 1; generalised stage Ⅰ: *n* = 5; localised stage Ⅱ: *n* = 6; localised stage Ⅲ: *n* = 8) groups. Comparison between the three groups revealed that patients with periodontitis were older than patients with gingivitis (*p* = 0.04). There was no significant difference in age between the periodontitis and healthy groups, and between the gingivitis and healthy groups. The distribution of sex between the three groups was not statistically significant. Table 1 shows the baseline characteristics of each group, with MGI, PI, BOP, PD, and CAL levels higher in the periodontitis and gingivitis groups than in the periodontal health group.

**Table 1.** Characteristics of the population and periodontal clinical parameters.

		Healthy (H)	Gingivitis (G)	Periodontitis (*P*)	*p* value
Age		27.0(26.0~35.5)	26.0(25.0~27.0)	37.0(25.5~50.5)	0.005^†^
Gender	Males	6(30%)	7(35%)	5(25%)	0.937^‡^
	Females	14(70%)	13(65%)	15(75%)	
MGI		1.27 ± 0.17	1.64 ± 0.27	1.85 ± 0.36	<0.001^†^
PI		0.70 ± 0.32	1.18 ± 0.37	1.06 ± 0.43	0.001^†^
BOP		4.75 ± 2.94	20.15 ± 8.28	20.90 ± 12.69	<0.001^†^
PD		1.44 ± 0.17	1.69 ± 0.18	1.95 ± 0.34	<0.001^†^
CAL		0	0	0.45 ± 0.27	<0.001^†^

^†^ Kruskal-Wallis test, the *p* value was adjusted by Bonferroni correction.

^‡^ chi-square test; MGI, modified gingival index; PI, plaque index; BOP, bleeding on probing; PD, probing depth; CAL, clinical attachment loss.

### GCF biomarkers and their association with clinical parameters

**Figure 1.**
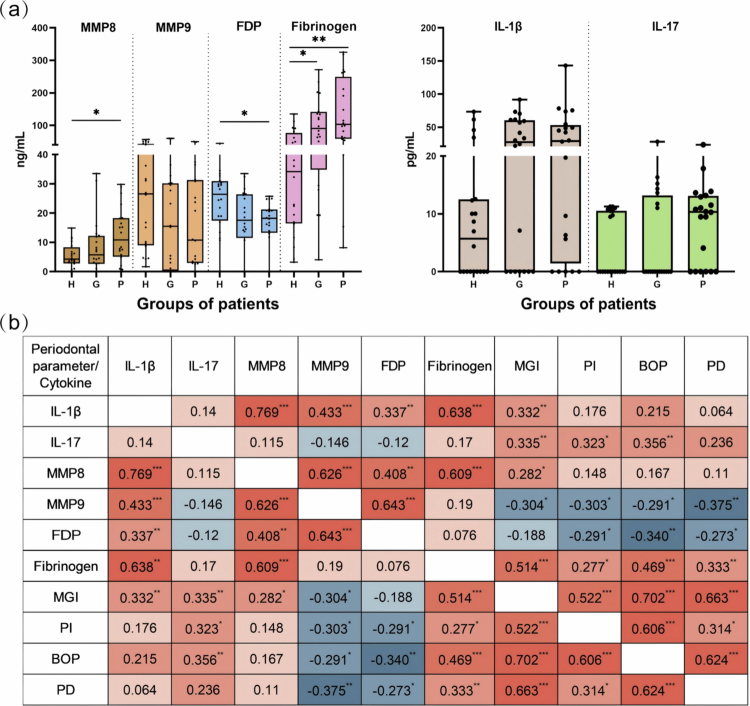
The Kruskal-Wallis test was used to compare inflammatory factors in GCF among the three groups (a); Spearman correlation coefficient matrix as a measure of the relationship between inflammatory factors and periodontal clinical parameters (b); Correlation values are translated into colour shades from blue (negative correlation) to red (positive correlation); H, healthy; G, gingivitis; *P*, periodontitis; IL-1β, interleukin-1β; IL-17, interleukin-17; MMP8, matrix metalloproteinase8; MMP9, matrix metalloproteinase9; MGI, modified gingival index; PI, plaque index; PD, probing depth; BOP, bleeding on probing; *, *p* < 0.05; **, *p* < 0.01.

Levels of fibrinogen, FDP, and MMP8 in GCF varied between the groups. Compared to the healthy group, fibrinogen was increased in the gingivitis group (*p* = 0.024) and the periodontitis group (*p* = 0.002). MMP8 levels were higher in the periodontitis group than that in the healthy group (*p* = 0.042).FDP in the healthy group was higher than that in the periodontitis group (*p* = 0.040). MMP9, IL-1β, and IL-17 levels showed no difference between the three groups (Figure 1a).

The correlation between GCF biomarkers and clinical parameters is shown in Figure 1b. Fibrinogen, IL-17, IL-1β and MMP8 were positively associated with clinical parameters. Fibrinogen and IL-17 were linked to increased MGI, PI, and BOP. Fibrinogen also had a strong positive correlation with PD (r_s_ = 0.333). IL-1β (r_s_ = 0.332) and MMP8 (r_s_ = 0.282) showed a significant positive correlation with MGI. MMP9 and FDP demonstrated negative correlation with clinical parameters. All the clinical parameters showed significant negative connections with MMP9 (*p* < 0.05). Apart from MGI, the remaining clinical parameters showed a negative correlation with FDP levels (*p* < 0.05).

### Association between the subgingival microbiome and clinical parameters

**Figure 2.**
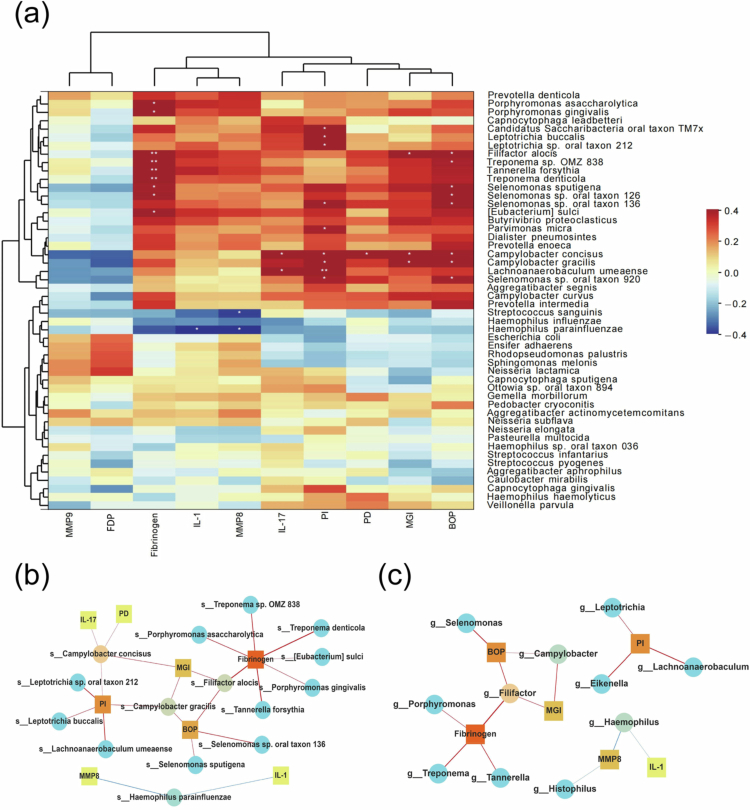
Multivariable Spearman correlation coefficient heatmap summarised the relationship among subgingival microorganisms, GCF cytokines, and periodontal clinical parameters at the level of the top 50 species (a); Reticulogram of species (b) and genera (c) in relation to cytokines and periodontal parameters, with line thickness representing the magnitude of correlation; *, *p* < 0.05; **, *p* < 0.01.

We investigated whether clinical parameters were related to the subgingival microbiome. Factors influencing the diversity of the subgingival microbiome were assessed. The relative abundance of the top 50 species was selected for Spearman correlation analysis with inflammatory biomarkers and periodontal parameters (Figure 2a). Factors that were statistically significant (*p* < 0.05) and had correlation coefficients >0.4 were plotted against microorganisms (species and genera) in a reticulation diagram (Figure 2b, 2c). Notably, more subgingival taxa showed significant associations with PI and BOP than with probing depth (PD) and MGI. At the genus level, *Lachnoanaerobaculum*, *Eikenella*, and *Leptotrichia* were all correlated with PI. *Campylobacter* and *Filifactor* were correlated with BOP and MGI. At the species level, *Campylobacter concisus* (*C. concisus*) was positively correlated with all the parameters, PI, MGI, BOP and PD (*p* < 0.05). *C. gracilis* was positively correlated with PI, MGI, and BOP (*p* < 0.05). *F. alocis* was positively correlated with BOP and MGI (*p* < 0.05). *Selenomonas sp. oral taxon 136* and *Selenomonas sp. oral taxon 920* were related to PI and BOP (*p* < 0.05).

**Figure 3.**
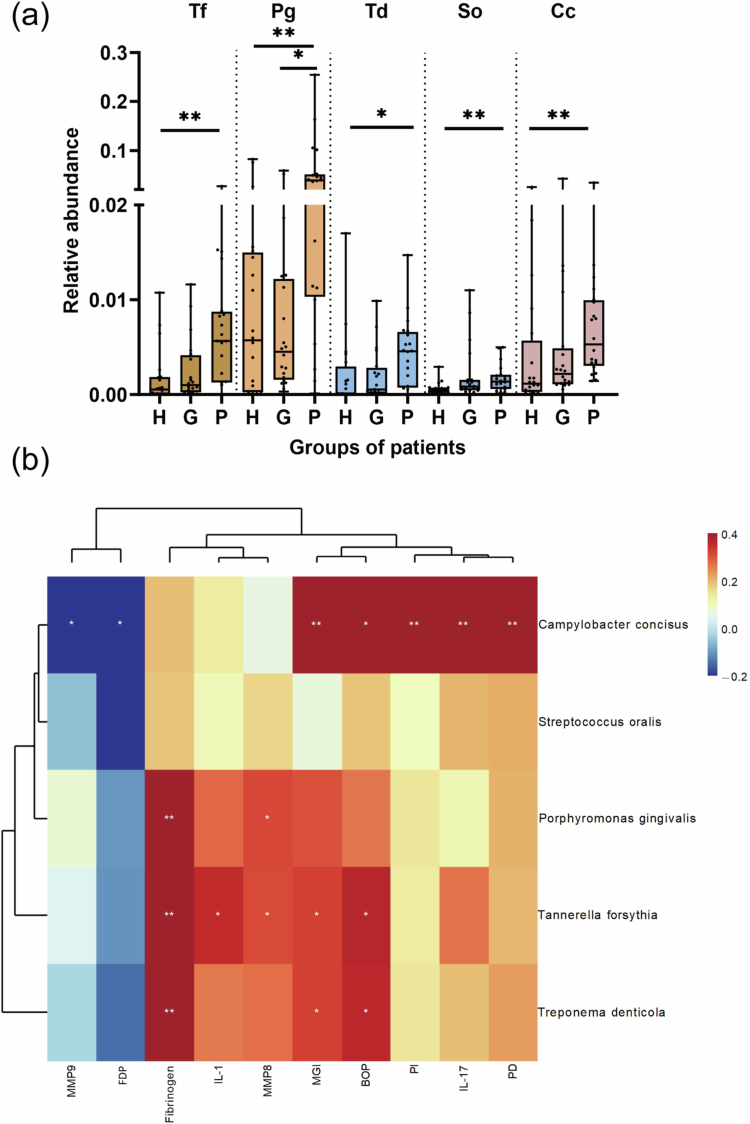
Comparison of the relative abundance of the red, orange, yellow, green, purple and blue complexes in healthy, gingivitis, and periodontitis groups, the Kruskal-Wallis test was used to compare the differences in microorganisms among the three groups (a); The heatmap shows the correlations among microorganisms, GCF biomarkers, and periodontal parameters (b); Tf, *T. forsythia*; Pg, *P. gingivalis*; Td, *T. denticola*; So, *S. oralis*; Cc, *C. concisus*; *, *p* < 0.05; **, *p* < 0.01.

We also analysed the red, orange, yellow, green, purple and blue complexes detected in the study. Figure 3 demonstrates the species with significant differences between groups. The relative abundance of *T. forsythia* (*p* = 0.005), *P. gingivalis* (*p* = 0.009), *T. denticola* (*p* = 0.014), *Streptococcus oralis* (*S. oralis, p* = 0.007), and *C. concisus* (*p* = 0.003) in the periodontitis group was significantly higher than the healthy group, and the relative abundance of *P. gingivalis* was significantly lower in the gingivitis group than in the periodontitis group (*p* = 0.013). Correlation analysis showed that the red complex (*T. denticola*, *T. forsythia*) was positively correlated with MGI and BOP. *C. concisus* showed a positive correlation with MGI, PI, BOP, and PD. No significant correlation was found between periodontal parameters and *P. gingivalis, S. oralis*.

### Association between inflammatory biomarkers and microbial taxa

To detect specific interactions, correlation analysis was performed between inflammatory biomarkers and subgingival microbial taxa (Figures 2, 3). Notably, fibrinogen was associated with much more microorganisms than the other inflammatory biomarkers. Fibrinogen showed significant positive correlations with *P. gingivalis*, *T. forsythia*, *T. denticola, F. alocis, Porphyromonas asaccharolytica* (*P. asaccharolytica*), *Treponema sp. OMZ 838, Eubacterium sulci* (*E. sulci*), *Selenomonas sputigena* (*S. sputigena*) and *Selenomonas sp. oral taxon 136*. Negative correlations were found between IL-1β (r_s_ = –0.43), MMP8 (r_s_ = –0.46) and *Haemophilus parainfluenzae* (*H. parainfluenzae*) A negative correlation was also shown between MMP8 and *Streptococcus sanguinis* (r_s_ = –0.38).

Among the periodontal pathogens, there was also a significant positive correlation between the red complex and fibrinogen (r_s_ = 0.42). MMP9 (r_s_ = –0.34) and FDP (r_s_ = –0.35) were negatively correlated with *C. concisus*. No significant correlation was found between inflammatory markers and *S. oralis*.

### Diagnostic accuracy analysis of inflammatory and microbial biomarkers

**Figure 4.**
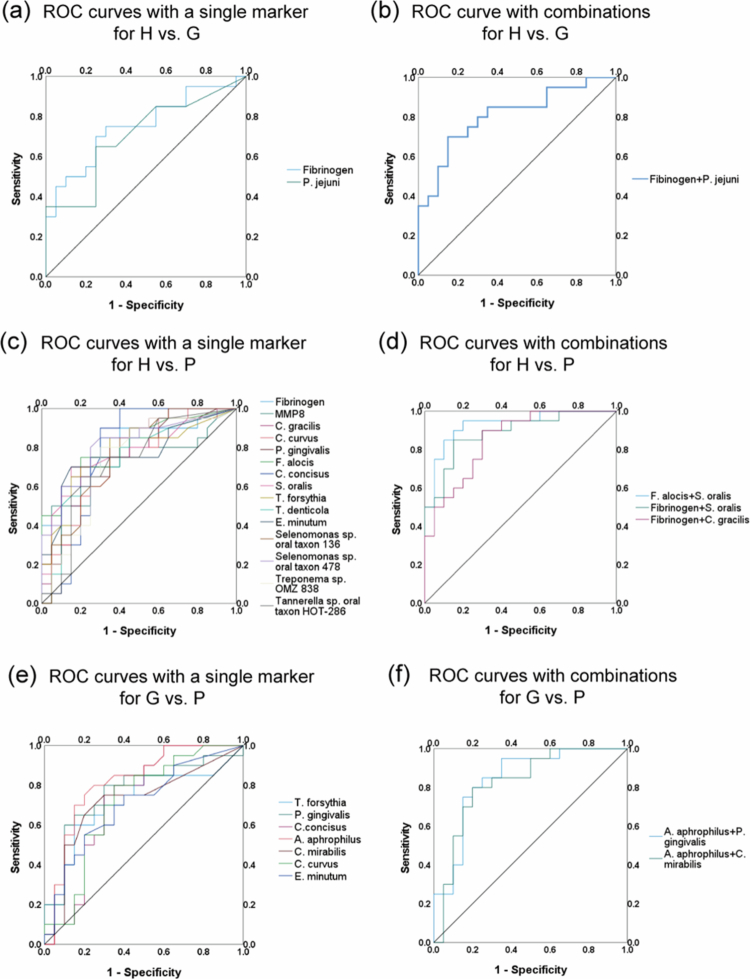
ROC curves for individual biomarkers and marker combinations to differentiate periodontal diseases; (a~b), H vs. G; (c~d), H vs. P; (e~f), G vs. P; H, healthy; G, gingivitis; *P*, periodontitis.

The biomarkers (inflammatory and microbial) might have the potential in differentiating periodontal disease due to their correlation with clinical parameters. The single markers and their combinations were analysed to differentiate between healthy and gingivitis, and healthy and periodontitis. Figure 4 displayed the markers and their combinations with the most significant diagnostic accuracy. Other markers with AUC ≥ 0.70 (Supplementary Table A) and marker combinations (Supplementary Table B) with AUC ≥ 0.85 are displayed.

Only fibrinogen (AUC = 0.752, *p* = 0.006) showed moderate diagnostic accuracy when differentiating between periodontal health and gingivitis. For the differentiation of periodontal health and periodontitis, *F. alocis* showed the highest accuracy in single marker (AUC = 0.821, *p* = 0.001), followed by fibrinogen (AUC = 0.81, *p* = 0.001). The combination of markers improved the diagnostic accuracy of periodontitis compared to single biomarkers. Among the combinations of two markers, the highest AUC value was found for the combination of *F. alocis* and *S. oralis* (AUC = 0.930, *p* < 0.001), fibrinogen also showed increased diagnostic accuracy in combination with *S. oralis* compared to the individual biomarkers (AUC = 0.888, *p* < 0.001). For the differentiation of gingivitis and periodontitis, *Aggregatibacter aphrophilus* (*A. aphrophilus*) showed the highest diagnostic accuracy (AUC = 0.807, *p* = 0.001), followed by *P. gingivalis* (AUC = 0.766, *p* = 0.005). The combination of *A. aphrophilus* and *P. gingivalis* demonstrated the highest diagnostic accuracy (AUC = 0.842, *p* < 0.001). No inflammatory markers of GCF were statistically significant in differentiating gingivitis from periodontitis.

## Discussion

The study found that higher levels of fibrinogen were detected in gingivitis and periodontitis. Among all the inflammatory cytokines, Fibrinogen was associated with all of the measured clinical parameters, BOP, MGI, PI, and PD. The highest correlation with specific subgingival microorganisms, e.g. *P. gingivalis*, *T. forsythia*, *T. denticola*, *F. alocis*, *P. asaccharolytica* , *Treponema sp. OMZ 838*, *E. sulci*, *S. sputigena* and *Selenomonas sp. oral taxon 136* were also shown in fibrinogen.

Elevated fibrinogen levels in the GCF are associated with periodontal inflammation. Gocke et al. demonstrated that patients with periodontitis had higher fibrinogen levels [26]. Our study found consistent conclusions, and fibrinogen was associated with more severe BOP, MGI, PI, and PD indices. This might related to the biological effects of fibrin (ogen). In periodontal diseases, fibrin aggregates at bleeding sites, mediating the coagulation response and inhibiting microbial transmission [27,28]. Excessive deposition of fibrin (ogen) may also facilitate pathogenic attachment and infection [29]. The binding of fibrin (ogen) to leucocytes regulates the activities of neutrophils, macrophages, and other inflammatory cells, inducing inflammatory effects such as reactive oxygen species damage and neutrophil extracellular traps [29–31]. The products fragment E and D dimers formed by fibrin hydrolysis are known to have pro-inflammatory effects [32].

Fibrinogen is associated with some subgingival microorganisms. Fibrinogen is thought to affect the colonisation of periodontal pathogens [33–35], which in turn influences the level of fibrinogen [36–38]. *T. forsythia* can interfere with coagulation by degrading fibrinogen and fibronectin through the secretion of mirolysin [37]. Arginine gingipain of *P. gingivalis* exerts a procoagulant effect, which leads to fibrin formation and the accumulation of extravascular fibrin causes chronic inflammation [36,38]. Differences between periodontal health and disease are often reflected in the red and orange complexes [39]. *C. gracilis* is a member of the orange complex and is thought to be associated with the progression of aggressive periodontitis, with a higher capacity to invade gingival fibroblasts than the red complex [40,41]. We found that *C. gracilis* was more abundant in the periodontitis group and was positively correlated with PI, MGI, and BOP. Differential changes in *C. gracilis* may reflect higher periodontal inflammation. As a newly discovered periodontitis-associated pathogen, *F. alocis* is also positively correlated with fibrinogen. High levels of *F. alocis* were positively correlated with BOP and MGI, further supporting the association between *F. alocis* and periodontitis. More research is required to investigate this relationship further.

Single or multiple biomarkers in saliva and GCF have been analysed for the correlation and diagnostic accuracy of periodontal diseases. *T. forsythia* in saliva was associated with further attachment loss (AUC = 0.647) [42]. The value of MMP8 as a marker for periodontitis has also been validated [43]. In addition, combinations of inflammatory biomarkers and different species of bacteria could be used to differentiate patients with periodontitis and improve detection sensitivity [44–47]. The analysis of GCF has been a widely utilised method for examining inflammatory processes associated with microorganisms [48]. We explored the diagnostic efficacy of inflammatory biomarkers and subgingival microorganisms in GCF. Compared with some well-established biomarkers, IL-1β, IL-17, MMP8, and MMP9, a more sensitive biomarker, fibrinogen was identified to be related to clinical parameters, and subgingival microorganisms. Moreover, fibrinogen serves as the sole GCF biomarker capable of distinguishing between healthy states and gingivitis. Fibrinogen also has an accuracy of 0.810, followed by *F. alocis* (AUC = 0.821) in the diagnosis of periodontitis. The diagnostic efficacy of periodontitis can be improved by combining individual biomarkers, with the combination of *F. alocis* and *S. oralis* demonstrating the highest diagnostic accuracy (AUC = 0.930, *p* < 0.001). *S. oralis* is an early coloniser of dental plaque that promotes biofilm growth through specific coaggregation with other pioneer species, thereby helping to seed biofilm development [49]. As early biofilms develop, streptococci can rapidly consume oxygen and contribute to anaerobic microniches, facilitating the establishment of obligate anaerobes [50]. Moreover, *Fusobacterium nucleatum* (*F. nucleatum*), a key ‘bridging’ organism, adheres to streptococci and supports integration into multispecies communities, and it can enhance the accumulation of *F. alocis* in in-vitro community models [51]. Given that *F. alocis* is increasingly recognised as a periodontal disease–associated organism, the joint detection of an early coloniser (*S. oralis*) and an emerging anaerobic periodontal pathogen (*F. alocis*) may better capture biofilm maturation and disease-associated community shifts, thereby improving diagnostic accuracy [52]. Periodontal parameters and radiographic examinations are routinely used for the diagnosis of periodontal diseases, but these examinations deeply rely on the expertise of examiners, and a proportion of indistinguishable patients often exists in real clinical situations [53].

The combination of fibrinogen and associated subgingival microorganisms may be helpful for discriminating periodontal diseases. With the global and national burden of periodontal diseases among older adults, patients and dentists face huge challenges when providing dental services [54]. The non-invasive testing of biomarkers may provide additional methods for disease monitoring and disease control to the patients.

This study had some limitations. The study selected limited biomarkers and had a small sample size.Due to the cross-sectional design, this study cannot explain the role of GCF and microbial markers in disease progression. Further longitudinal studies are necessary, particularly those involving the follow-up of patients with gingivitis and periodontitis after specific periodontal treatment (e.g. scaling and root planing), to evaluate the dynamic changes of these biomarkers and their prognostic value. Patients with periodontitis have healthy and diseased sites in the oral cavity, and inflammatory cytokines and microbial composition differ at different sites [14], this study could not exclude the influence of differences in sampling sites on the results. In addition, although this study illustrates the value of combined GCF and subgingival microorganisms in the diagnosis of periodontal disease, quantitative laboratory testing is time-consuming and costly to apply in daily dental practice, and more research is needed to explore clinical translational pathways and evaluate their effectiveness.

In conclusion, fibrinogen levels were higher in the GCF of participants with gingivitis and periodontitis, and numerous subgingival microorganisms and periodontal clinical parameters were associated with fibrinogen. Fibrinogen and its combination with *F. alocis* and *S. oralis* may indicate periodontal diseases. These findings should be further validated in longitudinal cohorts, particularly with pre- and post-treatment follow-up, to establish clinical utility and prognostic value.

## Supplementary Material

Table B.docx Table B.docx

Supplementary Material Table A.docx

## Data Availability

Reasonable requests for data about this study can be obtained from the corresponding author.
