# Supplementary material for: Fibrinogen was associated with subgingival microbiome in periodontal diseases: a pilot study
Source: J Oral Microbiol. 2026 Jun 2;18(1):2681264. doi: 10.1080/20002297.2026.2681264 (PMC13235256; doi:10.1080/20002297.2026.2681264)
Supplement: Table B.docx [file ZJOM_A_2681264_SM2429.docx]

**Table B** The diagnostic efficacy of combining markers to differentiate between healthy and periodontitis

|  | AUC | Se (%) | Sp (%) | YI | Optimal  threshold | *p* value |
| --- | --- | --- | --- | --- | --- | --- |
| *T. forsythia*+*S. oralis* | 0.907 | 95 | 80 | 0.75 | 0.376 | 0.000 |
| *E. minutum*+*S. oralis* | 0.905 | 95 | 75 | 0.7 | 0.249 | 0.000 |
| *Selenomonas sp. oral taxon 478*+*S. oralis* | 0.901 | 90 | 80 | 0.7 | 0.324 | 0.000 |
| *P. gingivalis*+*S. oralis* | 0.896 | 95 | 80 | 0.75 | 0.336 | 0.000 |
| *C. gracilis*+*S. oralis* | 0.885 | 90 | 80 | 0.70 | 0.392 | 0.000 |
| *Treponema sp. OMZ 838*+*S. oralis* | 0.864 | 85 | 80 | 0.65 | 0.411 | 0.000 |
| *T. denticola*+*S. oralis* | 0.861 | 80 | 85 | 0.65 | 0.471 | 0.000 |
| MMP8+ *S. oralis* | 0.858 | 80 | 95 | 0.75 | 0.560 | 0.000 |

Se, Sensitivity; Sp, Specificity; YI: Youden Index
