# Supplementary material for: Fibrinogen was associated with subgingival microbiome in periodontal diseases: a pilot study
Source: J Oral Microbiol. 2026 Jun 2;18(1):2681264. doi: 10.1080/20002297.2026.2681264 (PMC13235256; doi:10.1080/20002297.2026.2681264)
Supplement: Supplementary Material — Table A.docx [file ZJOM_A_2681264_SM2430.docx]

**Table A** The diagnostic efficacy of single markers in differentiating between healthy and periodontitis, and between gingivitis and periodontitis.

|  |  | AUC | Se(%) | Sp(%) | YI | Optimal  threshold | *p* value | PPV(%) | NPV(%) |
| --- | --- | --- | --- | --- | --- | --- | --- | --- | --- |
| H vs. P | *Selenomonas sp. oral taxon 478* | 0.809 | 87 | 70 | 0.55 | 0.0006 | 0.001 | 73.9 | 82.4 |
|  | *R. pickettii* | 0.778 | 90 | 60 | 0.5 | 0.0003 | 0.003 | 69.2 | 85.7 |
|  | *S. oralis* | 0.774 | 70 | 85 | 0.55 | 0.001 | 0.003 | 82.4 | 73.9 |
|  | *C. concisus* | 0.772 | 90 | 70 | 0.6 | 0.002 | 0.003 | 75.0 | 87.5 |
|  | *C. gracilis* | 0.770 | 70 | 80 | 0.5 | 0.020 | 0.003 | 76.5 | 69.6 |
|  | *E. minutum* | 0.768 | 70 | 85 | 0.55 | 0.0011 | 0.004 | 77.8 | 72.7 |
|  | *P. gingivalis* | 0.766 | 65 | 85 | 0.5 | 0.016 | 0.004 | 81.3 | 70.8 |
|  | *T. forsythia* | 0.765 | 85 | 65 | 0.5 | 0.001 | 0.004 | 70.8 | 76.5 |
|  | *Selenomonas sp. oral taxon 136* | 0.758 | 80 | 70 | 0.5 | 0.0007 | 0.005 | 66.7 | 84.6 |
|  | *T. denticola* | 0.741 | 70 | 75 | 0.45 | 0.002 | 0.009 | 72.2 | 68.2 |
|  | *Treponema sp. OMZ 838* | 0.736 | 80 | 70 | 0.5 | 0.0049 | 0.011 | 71.4 | 73.7 |
|  | *Arachidicoccus sp. KIS59-12* | 0.734 | 90 | 50 | 0.4 | 0.0003 | 0.011 | 64.3 | 83.3 |
|  | *MMP8* | 0.731 | 70 | 75 | 0.45 | 6.967 | 0.012 | 73.7 | 71.4 |
|  | *C. curvus* | 0.729 | 80 | 65 | 0.45 | 0.0045 | 0.013 | 69.6 | 76.5 |
|  | *Tannerella sp. oral taxon HOT-286* | 0.725 | 75 | 65 | 0.4 | 0.0003 | 0.015 | 68.2 | 72.2 |
| G vs. P | *C. mirabilis* | 0.719 | 75 | 70 | 0.45 | 0.0001 | 0.018 | 59.1 | 61.1 |
|  | *C. curvus* | 0.714 | 80 | 65 | 0.45 | 0.0043 | 0.021 | 69.6 | 76.5 |
|  | *T. forsythia* | 0.709 | 60 | 84.2 | 0.442 | 0.005 | 0.025 | 80.0 | 68.0 |
|  | *C. concisus* | 0.707 | 75 | 68.4 | 0.434 | 0.003 | 0.027 | 68.2 | 72.2 |
|  | *E. minutum* | 0.705 | 75 | 60 | 0.35 | 0.0005 | 0.027 | 65.2 | 70.6 |

Healthy and Periodontitis (H vs. P); Gingivitis and Periodontitis (G vs. P); Se, Sensitivity; Sp, Specificity; YI: Youden Index; PPV: positive predictive value; NPV: negative predictive value
